# Safety and Effect of Bariatric Metabolic Surgeries for Psychiatric Patients with Obesity: A Retrospective Matched Case–control Trial

**DOI:** 10.1007/s11695-023-06627-x

**Published:** 2023-05-13

**Authors:** Mohamed Hany, Mohamed Fahmy Aboudeeb, Clara Shapiro-Koss, Ann Samy Shafiq Agayby, Bart Torensma

**Affiliations:** 1 Department of Surgery, Medical Research Institute, Alexandria University, 165 Horreya Avenue, Hadara, 21561 Alexandria Egypt; 2 Consultant of Bariatric Surgery at Madina Women’s Hospital, Alexandria, Egypt; 3 Psychiatry Clinic, Madina Women’s Hospital, Alexandria, Egypt; 4 Psychiatry Clinic, GGZ-Delfland, Delft, The Netherlands; 5 Clinical Epidemiologist, Leiden University Medical Center (LUMC), Leiden, The Netherlands

**Keywords:** Bariatric metabolic surgery, Psychiatric illness, Incidence, Safety, Efficacy

## Abstract

**Introduction:**

Patients living with psychiatric illnesses (PIs) have a high prevalence of obesity. In a 2006 survey, 91.2% of professionals in the bariatric field identified “psychiatric issues” as clear contraindications to weight-loss surgery.

**Methods:**

This retrospective matched case–control study investigated the impact, safety, and possible relapse after bariatric metabolic surgery (BMS) in patients with PIs. Also, we tested the incidence of patients who developed PI after BMS and compared the post-procedural weight loss with that in a matched control group without PIs. The cases were matched in a ratio of 1:4 to the control patients standardized for age, sex, preoperative BMI, and type of BMS.

**Results:**

Of 5987 patients, 2.82% had a preoperative PI; postoperative de novo PI was present in 0.45%. Postoperative BMI was significantly different between the groups when compared to preoperative BMI (*p* < 0.001). Percentage of total weight loss (%TWL) after six months was not significantly different between the case (24.6% ± 8.9) and control groups (24.0% ± 8.4, *p* = 1.000). Early and late complications were not significantly different between the groups. The psychiatric drug use and dosage changes did not differ significantly pre- and postoperatively. Of the psychiatric patients, 5.1% were postoperatively admitted to a psychiatric hospital (*p* = 0.06) unrelated to BMS, and 3.4% had a prolonged absence from work after surgery.

**Conclusion:**

BMS is an effective weight loss treatment and a safe procedure for patients with psychiatric disorders. We found no change in the patients’ psychiatric status outside the usual disease course. Postoperative de novo PI was rare in the present study. Furthermore, patients with severe psychiatric illness were excluded from undergoing surgery and, therefore, from the study. Careful follow-up is necessary to guide and protect patients with PI.

**Graphical Abstract:**

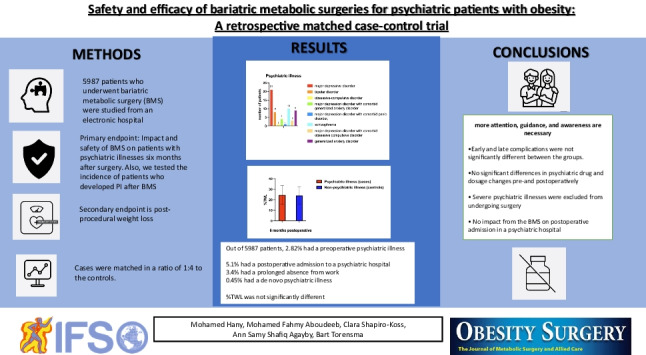

## Introduction

Patients with psychiatric illnesses (PIs) have a high prevalence of obesity, nearly double that of the general population [1, 2]. Non-surgical treatments and pharmacological interventions showed almost no effect on weight loss (WL) in patients with PI, with inconsistent study data [3–7].

A broad spectrum of psychiatric disorders falls under various psychiatric diagnoses. Different psychiatric diagnoses, such as psychosis, and mood and anxiety disorders, involve different behaviors and have different treatment pathways. Some of the most common diagnoses include the major depressive disorder (MDD), bipolar disorder, obsessive–compulsive disorder (OCD), MDD with a comorbid generalized anxiety disorder (GAD), MDD with comorbid panic disorder, schizophrenia, MDD with comorbid OCD, and GAD, as described in the Diagnostic and Statistical Manual of Mental Disorders (DSM-5) [8].

Several studies have found an association between obesity and PI, and a higher prevalence of PI in patients with severe obesity seeking bariatric metabolic surgery (BMS). The focus has been mainly on antipsychotic drug-induced weight gain and schizophrenia [9, 10]. Another study found that BMS effectively reduced psychiatric patients’ body weight and improved their quality of life, especially in those with severe depressive, bipolar, and schizoaffective disorders [11]. In addition, patients with bipolar disorders who underwent Roux-en-Y gastric bypass (RYGB) had successful weight loss outcomes at 12 months, which did not significantly differ from those of patients without PIs [12].

In a 2006 survey, 91.2% of professionals in the BMS field identified “psychiatric issues” as clear contraindications to weight loss surgery [13]. Research on the effects of BMS in patients with various PIs is lacking. Individual PI diagnoses within the BMS have been investigated, but not the effect on the overall prevalence of BMS and the effect of multiple PI diagnoses. Furthermore, since the above survey from 2006, no new surveys in the BMS field have been conducted; therefore, further research is necessary to produce clear outcomes on the impact of BMS on patients with PIs.

This study aimed to test the safety, efficacy, impact, and possible relapse after BMS in patients with a broad spectrum of PIs, whereby changes in medication, type and dosage, admission, or prolonged absence from work were tested. Also, we tested the incidence of patients who developed PI after BMS. The secondary objective was to compare postprocedural weight loss with a matched control group without PI.

## Methods

This retrospective matched case–control trial used records from an electronic hospital database from August 2018 to March 2022 of patients with preoperative PIs who were compliant with psychiatric medications and were followed up for six months at Madina Women’s hospital and Medical Research Institute, Alexandria University, Egypt. All patients were assessed preoperatively by a multidisciplinary team (MDT), including bariatric surgeons, nutritionists, internists, and psychiatrists.

### Study endpoints

The primary endpoint was to test the impact, safety, and possible relapse into psychiatric illness after BMS in patients with preoperative PIs within six months after surgery. Also, we tested the incidence of patients who developed PI after BMS.

The secondary endpoint was post-procedural weight loss and complications compared with a matched control group without PI.

### Inclusion and exclusion criteria

Patients were aged between 18 and 60 years, with current active-controlled PIs defined according to the DSM 5 criteria [8], no associated medical conditions, and no substance abuse. Patients with a severe psychiatric diagnosis specifier were deemed unsuitable for surgery and were excluded.

### Relapse

Relapse was defined as hospital admission or prolonged absence from work.

### Postoperative consultations with psychiatry outpatient clinics

The patients were required to visit the clinic psychiatrist after two weeks and recommended to undergo another visit in the first month and two visits a month for up to six months postoperatively. A total of eleven visits were possible.

### Data collection

We monitored the patients’ psychiatric conditions during the first six postoperative months. Relapse was defined as: (1) the need to increase the dose of current psychiatric medications, (2) switching to a different type of psychiatric medication, (3) prolonged absence from work, and (4) admission to a psychiatric hospital.

Baseline characteristics were collected, including age, sex, BMI, and type of surgery.

Postoperatively, the percentage of total weight loss (%TWL) was calculated using the following formula: %TWL = ([initial body weight – current body weight]/ initial body weight) × 100%. The early and late complications after BMS were recorded.

## Surgical technique

All BMS were performed according to recommendations from the International Federation for the Surgery of Obesity and Metabolic Disorders (IFSO), and World Consensus Meeting statements [14, 15]. Two independent surgeons who operated on over 800 patients per year performed all the procedures.

### Statistical methods

Descriptive and inferential statistics were used for the analyses. All data were first tested for normality using the Kolmogorov–Smirnov test, the Q–Q plot, and Levene’s test. Categorical variables are expressed as n (%). Continuous normally distributed variables are expressed as means and standard deviations, and non-normally distributed data are expressed as medians and interquartile ranges for skewed distributions. When analyzing group data, categorical variables were tested using Pearson’s chi-square test or Fisher’s exact test as appropriate. Normally distributed continuous data were tested with dependent samples using the Student’s *t*-test for pre and postoperative results. For skewed (non-parametric) data, the Wilcoxon signed-rank test was used. The cases were matched to the controls at a 1:4 ratio; the matched variables included age, sex, preoperative BMI, and type of BMS. No replacements of the controls were applied. Statistical significance was set at *p* < 0.05. Statistical analyses were performed using the R (version 4.0.4) package.

### Post-hoc-power analysis

A post-hoc-power analysis with G*power version 3.1.9.5 was used for post-hoc sample size calculation for this study's presented PI prevalence of 2.82%. With a power of 0.8 and an alpha of 0.05, a study size of 276 patients was found to be necessary to detect PIs in a cohort.

## Results

This was a retrospective matched case–control trial wherein the electronic medical records of 5987 patients who underwent BMS were screened, revealing 169 (2.82%) patients with a preoperative psychiatric illness, 59 of whom agreed to participate in this study.

After matching the cases with controls for age, sex, preoperative BMI, and type of BMS, there were no significant differences between the groups (*p* = 1.000). Of the patients with PIs, 45 (76.2%) were female, and the age in years was mean ± SD, 36.9 ± 15.4. The conducted bariatric procedures were laparoscopic sleeve gastrectomy, 52 (88.1%), and Roux-en-Y gastric bypass, 7 (11.9%) (Table 1).

**Table 1:** Baseline demographics *(Matched case–control 1:4 ratio, no replacements)*

	Psychiatric illness *N* = 59 (cases)	Non-psychiatric illness *N* = 236 (controls)	*P* value
Sex (female) n (%)	45 (76.2%)	180 (76.3%)	1.000
Age (in years) mean ± sd	36.9 ± 15.4	36.4 ± 13.9	1.000
Height (in cm) mean ± sd	165.9 ± 10.2	165.4 ± 9.8	1.000
Weight (in kg) mean ± sd	129.5 ± 27.1	128.7 ± 26.9	1.000
Preoperative BMI (kg/m^2^) mean ± sd	47.7 ± 10.5	47.3 ± 9.4	1.000
Postoperative weight at 6 months (in kg) mean ± sd	97.0 ± 20.5	96.4 ± 19.7	1.000
Postoperative BMI at 6 months (kg/m^2^) mean ± sd	35.9 ± 7.4	35.6 ± 6.8	1.000
%TWL at 6 months mean ± sd	24.6 ± 8.9	24.0 ± 8.4	1.000
Bariatric procedures n%			
Laparoscopic sleeve gastrectomy n (%)	52 (88.1)	207 (87.7%)	1.000
Roux en Y Gastric Bypass n (%)	7 (11.9)	29 (12.3%)	
Early complications n (%)			
VTE	1 (1.7%)	5 (2.1%)	1.000
Pneumonia	2 (3.4%)	6 (2.5%)	0.874
Melena	1 (1.7%)	7 (2.9%)	0.912
Abdominal bleeding	0 (0.0%)	2 (0.8%)	0.864
Wound infection	0 (0.0%)	1 (0.4%)	1.000
Late complications n (%)			
Port site hernia	2 (3.4%)	9 (3.8%)	1.000
Internal herniation	1 (1.7%)	0 (0.0%)	0.946
CCC	0 (0.0%)	12 (5.1%)	**0.04**

*p*-values are significant ≤0.05

%*TWL*: percentage total weight loss, *VTE*: Venous thromboembolism, *CCC*: Concomitant cholecystectomy

## PI in BMS

### Preoperative diagnoses

The psychiatric disorders were distributed as follows: MDD, 35.6%; bipolar disorder, 13.6%; OCD, 1.7%; MDD with comorbid GAD, 10.2%; MDD with comorbid panic disorder, 1.7%; schizophrenia, 16.9%; MDD with comorbid OCD, 5.1%; GAD, 15.4%.

### Preoperative medications

Of the included patients, 30 used anti-depressants (50.8%), nine used antipsychotic agents (15.3%), eight used anti-depressants + antipsychotic agents (13.6%), one used a mood stabilizer (1.7%), two used anti-depressants + mood stabilizers (3.4%), four used antipsychotic agents + mood stabilizers (6.8%), two used benzodiazepines (3.4%), and three used anti-depressants + benzodiazepines (5.1%).

### Postoperative changes

#### Relapse

Of the included patients, three (5.1%) were postoperatively admitted to a psychiatric hospital (one with depression, one with depression + GAD, and one with schizophrenia) (p = 0.06). Of the three patients, two had a history of psychiatric hospital and postoperative admissions, however, these were unrelated to the BMS (one patient had depression and the other had schizophrenia) (p = 0.375).

Two (3.4%) patients experienced prolonged absence from work after surgery (one with depression and one with schizophrenia).

Of all the patients, 13.6% had a history of admission to a psychiatric hospital (three with depression, two with bipolar disorder, and three with schizophrenia), but were stable when BMS was scheduled.

#### Drug changes

Patients were monitored during the postoperative phase and medication changes were recorded. Six patients (10.2% of the total cohort) had their antidepressant medication doses increased, four (6.8%) received an extra half dose, and in two (3.4%), the dose was increased once (*p* = 0.755). Patients on other drugs did not require a dose increase.

The dose was decreased in 12 patients (20.3%). In ten patients (16.9%), their antidepressant medication was decreased, in one (1.7%), an anxiolytic, and in one (1.7%), an antipsychotic agent (*p* = 0.224). Patients on other drugs did not require a dose decrease.

The drug was changed in 11 (18.6%) patients. In five (8.5%), another anti-depressant was used; in one (1.7%), an antipsychotic agent was added to the antidepressant; in one (1.7%), a mood stabilizer was changed to an antipsychotic agent; and in four (6.8%), another antipsychotic agent was registered (*p* = 0.692).

#### Number of consultations in the psychiatric outpatient clinic

A median (min–max) of 11 (0–11) visits was recorded in 6 months. Three patients (5.1%) did not visit the clinic at all. Three patients (5.1%) had two missed visits, and five (8.5%) missed one visit in the 6 months of follow-up (*p* = 0.341). When the patients were not attending to visit a psychiatrist at the clinic, the clinic kept attempting to contact the patient through various channels, including phone, email, and WhatsApp, for several months. If no contact was possible, the family was reached and kept track of the patient's well-being. In case of any changes or deterioration in the patient's PI condition, the family could approach the clinic again for psychiatric help. However, none of the patients who missed the postoperative visits had a relapse, and three had their medication dose decreased.

#### De novo psychiatric disorder

Of the total cohort without preoperative PI diagnoses, 27 patients (0.45%) developed de novo PI. Twenty-one patients (77.8%) had depression, two (7.4%) had adjustment disorders, two (7.4%) had personality disorders, one (3.7%) had a panic disorder, and 1 (3.7%) had a somatic symptom disorder (*p* = 0.001 compared the PI group). Twenty-one (77.8%) patients required psychiatric medication and six (22.2%) received psychotherapy sessions. There were no (0.0%) psychiatric admissions or prolonged absence from work. A median (min–max) of 11 (0–11) visits over 6 months was recorded. None of the patients (0%) did not visit the clinic. Two (7.4%) had 10 missed visits, two (7.4%) had two missed visits, and three (11.1%) missed one visit during the 6-month follow-up (*p* = 0.743).

#### BMI

Preoperative BMI (mean ± SD) was 47.3 ± 10.5 in the PI group, and 47.3 ± 9.4 in the control group (*p* = 1.000). Patients with PIs lost even amount of weight after six months than those in the control group. Postoperative BMI at six months was not significantly different between the two groups (PI [35.3 ± 7.4 and non-PI 35.6 ± 6.8] [*p* = 1.000]]. BMI was significantly reduced postoperatively in both groups (*p* < 0.001). The percentage of total weight loss (%TWL) after six months was 24.6 ± 8.9 in the PI group and 24.0 ± 8.4 in the control group (*p* = 1.000) (Table 1).

#### Complications

Early complications in the case group were one venous thromboembolism (VTE) (1.7%) compared with five (2.1%) in the control group (*p* = 1.000). Pneumonia occurred in two patients with PI (3.4%) and in six (2.5%) control patients (*p* = 0.874), melena in one patient with PI (1.7%), and in seven (2.9%) control patients (*p* = 0.912), no abdominal bleeding in patients with PI and in two (0.8%) control patients (*p* = 0.864), and no wound infection in patients with PI and in one (0.4%) control patient (*p* = 1.000).

Late complications were two (3.4%) post-site hernias in patients with PI and nine (3.8%) in the control group (*p* = 1.000), one internal herniation (1.7%) in a PI patient and none in the control group (*p* = 0.946), no concomitant cholecystectomy cases in the group with PI, and 12 (5.1%) cases in the control group (*p* = 0.04) (Table 1).

## Discussion

This retrospective study matched preoperative PI patients who were compliant with their psychiatric medications followed up for six months and controlled with patients without PIs. The primary endpoint was to test the impact, safety, and possible relapse into psychiatric illness after BMS in patients with PIs within six months after surgery. Also, we tested the incidence of patients who developed PI after BMS.

The secondary endpoint was post-procedural weight loss compared with a matched control group without PI.

In total, 2.82% patients in the available database were identified as having a PI, and 0.45% had de novo PI. Of the patients with preoperative PI, 5.1% were postoperatively admitted to a psychiatric hospital (one with depression, one with depression + GAD, and one with schizophrenia) (*p* = 0.06), and 3.4% had prolonged absence from work after the operation (one with depression and one with schizophrenia). Furthermore, both groups had a postoperative BMI at six months that did not significantly differ.

It is widely assumed that BMS can cause stress that in turn predisposes psychiatric patients to relapse. Owing to this assumption, there are limited data on psychiatric patients undergoing BMS.

### Differences in prevalence

The prevalence of PI in the general population is 12.5% [16], whereas we found it to be 2.82% in the present study of patients with obesity. Since every patient is screened in our clinic for any form of psychiatric disorder, this could be a realistic representation of the actual prevalence in our cohort; however, an underestimate of the prevalence in the general population.

For example, a national survey in 2017 in our country, Egypt, found a 10.75% prevalence of depression in the general population. However, only 0.4% of the patients were actively being treated for their disorder [17]. The US National Health and Nutrition Examination Survey from 2015 to 2018 found 7.2% of depression, and in general a 13.2% usage of anti-depression medication [18]; this means a 33-fold greater use of antidepressant medication. This could reflect in the amount of screening and treatment of patients who are undergoing BMS in both countries and could affect the postoperative changes in medication, if necessary. This shows that there is much disparity between countries in terms of medication use in patients with PI.

An older study by Sarwer et al. from 2005 showed that in patients who underwent BMS, a much higher prevalence (20%–60%) suffered from psychiatric disorders (e.g., depression, anxiety disorder, bipolar affective disorder, autism, phobias, schizophrenia) and, based on DSM-IV criteria, up to 72% of those who underwent BMS had personality disorder diagnoses than in our study [19].

Consequently, this would mean that two to six out of ten patients operated on had a psychiatric disorder. However, when profoundly assessing the studies in Sarwer et al., seven references were used for the statement of the PI in that study. The studies included only 57–153 patients for diagnosing PI. Most studies selected a group of patients from hospital records to test the diagnoses. This raises questions about selection bias.

Additionally, we could only discuss the power of the study in detecting psychiatric disorders in a range of 57 to 153 patients and have used it as the prevalence number. All psychiatric disorders can have various etiologies and can manifest under different diagnoses, all of which comprise specific effects and require specific therapies. Nevertheless, most studies presented their results only as the effect of “percentage of patients with a psychiatric disorder,” which can cause misleading interpretations. For example, one study only tested the binge eating diagnosis in 100 patients by way of an interview and found that 40% of patients indulged in binge eating; this was noted as a psychiatric disorder, which is correct, however, it is also a clear exclusion criterion for BMS. This has led to the wrong expression (“40% had a psychiatric disorder”) being used when cited in other studies as “40% of the patients had PI.” Another study tested 153 patients, 58% of whom had possible disorders; however, a different result in the same study concluded that 9% of patients had real PI. Nevertheless, 58% was used as the disorder percentage, but this was an inaccurate representation and therefore an overestimate of the real percentages.

In conclusion, we have no doubts regarding the results presented in the studies, but making a statement on the prevalence of PI would require additional statistical power in terms of sample size and extensive research of the full range of PI disorders. Our study has a reasonable prevalence adjustment since we tested all the patients from 5987 records.

A study by Vermeer et al. in 2020 tested all the BMS patients in one year (2015) with DSM IV axis 1 or 2 diagnoses, and used the remaining patients from the same year as controls [20]. A total of 2525 were operated, and 163 had a PI. This is a prevalence of 6.5%, which is more realistic and closer to that of our cohort (2.82%).

Furthermore, a possible explanation for the differences in prevalence can come from the misconceived advice against BMS given by general psychiatrists, as they may still think that surgery will induce a relapse; this explains the low incidence of patients in our study.

Since research on patients with PI is limited, creating guidelines for BMS remains difficult.

Psychiatric issues have been identified as clear contraindications to weight loss surgery [13], no new surveys on the opinions of BMS professionals regarding this issue have not been performed.

The recent 2022 guidelines by IFSO included a short paragraph dedicated to the treatment of patients with mental health issues, in which it is stated that licensed mental health providers with specialized knowledge and experience in BMS behavioral health need to assess the candidates for psychopathology and determine their ability to cope with the adversity of surgery and the changes in body image and lifestyle after BMS [21]. Despite this, proper in-depth guidelines on this matter are lacking when compared to all the other available knowledge on other associated medical problems in BMS.

In a more recent study from 2017 by Pearl et al., it is stated that mental health professionals vary widely in their criteria for determining whether a patient is an “acceptable” candidate for BMS [22].

Another study by Kiser et al. from 2023 noted a deficit of recent evidence regarding the association of diagnoses with the decision to require a follow-up after psychological evaluation (i.e., no required follow-up, required follow-up, placed on hold). There was also a variation in the required follow-up and hold rates between two BMS clinics (15% vs. 28%) [23].

Therefore, as recommended in the guidelines from the IFSO, experience in the field of BMS is essential, and knowledge specific to the BMS field is increasing. In our opinion, there is a lack of awareness of these diseases in well-conducted studies on PI outcome with attention to increasing statistical power to detect the realistic prevalence of PI, relapse, and safety, since this is still underrepresented in the current literature. However, the IFSO guidelines are not sufficiently clear.

### BMS and PI

A qualitative study found that patients with severe psychiatric issues benefited from BMS, which improved their quality of life and physical and mental health. The study tested a broad spectrum of PI diagnoses, including severe depressive disorder, bipolar disorder, and schizoaffective disorder, and found that patients self-requested extra care and support for obesity treatment and aftercare [11]. Therefore, allowing extra time, attention, and listening to the patient can help improve mental health outcomes in the postoperative period.

A 2020 review on the psychiatric management of patients who underwent BMS assessed 37 studies. Their results suggested that psychiatric medications do not negatively impact weight loss or health-related quality of life after BMS in the short term. However, more research on how specific psychiatric drugs and individual PIs affect long-term weight loss and psychosocial outcomes would be beneficial [24].

Our study showed no significant changes in medication dosages postoperatively, and patients with PI did not have significantly prolonged absences or readmissions to psychiatric hospitals. None of the readmissions were related to BMS. In our study, 59 patients with eight different diagnoses were included, who showed no significant worsening of symptoms or relapses outside the natural course of their diseases. This suggests that there the surgery had no significant impact on their PI’s.

A study by Gordan et al. found that self-esteem was more important than BMI regarding changes in depression and binge eating symptoms. When considering BMS, the priority is weight loss; however, in patients with PIs, variables other than weight loss may impact the perceived outcomes. Another study also used the BMI as the benchmark for assessing the improvement after BMS; however, they found that weight loss alone was not the main criterion for an improved BMS outcome in patients with a psychiatric background [25]. Therefore, the awareness and focus in PI patients in terms of life improvement is less focused on weight, and more on the other previously mentioned factors. Despite these findings and concerns, in a study by Mitchell et al., BMS improves depressive symptoms and was significantly associated with changes in BMI [26]. Therefore, weight loss is effective and linked to improving PI, but for patients with PI, it is less prominent, and this is something to consider for the postoperative phase and treatment.

Our study assessed the six-month period following surgery, as it is the one during which the greatest changes occur regarding weight, eating habits, lifestyle, and patient psychological health. This approach has been used in several studies [27–29]. A prolonged observation period may make it more difficult to determine whether changes are correlated with the operation or with additional external factors and events.

When we assessed the literature on follow-up longer than 6 months and on the adverse mental health outcomes after BMS, fewer studies have been published on relapse and the status of different PI diseases. In particular, the quality of life (QoL) outcome has been published, as seen in a recent systematic review by Jumbe et al. on the psychosocial QoL. Eleven studies showed that BMS is more effective in the long term for overall QoL than non-surgical treatments, with modest psychosocial benefits. In addition, significant improvements in psychosocial and physical QoL have been observed after a 2-year follow-up after BMS compared to non-surgical interventions. Only for results from follow-ups over 10 years showed minimal improvements in psychosocial QoL compared to non-surgical interventions. No information was presented regarding the relapse or specific PI diagnoses [30].

A study on specific PI diagnoses, depression, and anxiety, a systematic review by Gill et al. in 2019 showed that in 93% of the reported studies, there was a significant similar reduction in symptom severity 2–3 years after BMS, and that there were reductions in overall anxiety symptom severity after ≥ 24 months of follow-up after BMS. Preoperative anxiety and depression scores did not predict postoperative BMI. Similarly, post-surgery weight loss did not predict changes in anxiety symptoms [31].

A recent study from Vermeer et al. in 2020 examined health-related QoL (HRQoL). The study tested patients with PI and compared them with a control group named the generic group. Improvement in personal health status was significantly higher in the generic group from baseline at 12 months (*P* = 0.002), 24 months (*P* = 0.0018), 36 months (*P* = 0.025), and 48 months of follow-up (*P* = 0.003). Therefore, patients with PI had worse QoL after surgery than those in the control group. Nevertheless, the change in mental health-related QoL significantly differed between the baseline and the 48-month follow-up (*P* = 0.014), therefore, the PI patients improved compared to their preoperative status [20].

Therefore, our study could help elucidate the effects of relapse and medication changes in the first 6 postoperative months, as this was not published in other studies. A longer follow-up is necessary to understand the impact of BMS on PI patients. It is important to highlight that the patients in our database were only included if they were fit for surgery, i.e., had a stable mental illness, the stability of which was stability tested by a multi-disciplinary team. Owing to ethical implications, no research has been conducted on the outcomes of patients with unstable PIs undergoing BMS.

### Post hoc power analysis

Since PIs are widely discussed in the literature, their actual effects need to be clarified, especially in patients with obesity. Our post hoc power analysis showed a prevalence of 2.85%, suggesting that 276 patients would need to be included to accurately detect PIs. Our study identified 169 patients with PIs (without the 107 patients who declined study inclusion). Had they been included, the prevalence would have increased, and the power analysis would have shown a lower number.

The knowledge of a precise representation of the prevalence of PIs in patients with obesity could help us better understand this problem and improve patient care. The difference in sample numbers, from 169 to 59 patients, was due to patients opting not to participate. Patients who experienced relapse were primarily seen in the clinic as they required treatment. In contrast, patients who felt well and required no drug changes were less willing to participate and wanted to continue their everyday lives. Therefore, it could be expected that the patients who opted not to participate did not perform worse.

Therefore, more attention, guidance, and awareness should be given to individual patients with psychiatric disorders rather than a possible upfront refusal. Refusal to perform surgery due to PI can adversely affect patients' health in the long term. Disorder-specific research is required to determine the impact of the BMS on patients with each particular psychiatric condition. Research on psychiatric strategies in the bariatric community is necessary to better understand the needs of psychiatric patients.

### Safety after BMS in patients with PIs

Fink et al.'s 2021 systematic review investigated the impact of BMS on patients with MDD, anxiety disorder, bipolar disorder, schizophrenia, alcohol and drug use, and unspecified psychiatric histories. All evidence found was low or very low in quality, and recommendations were weak, highlighting the importance of well-conducted research involving patients with PI. The recommendation for patients with MDD is to monitor patients with a lifetime history of depression involving suicidality, psychiatric admission, and antidepressant use, for psychological complications during postoperative recovery. For severe mental illnesses, patients with a lifetime history of bipolar disorder or schizophrenia should always be monitored following BMS by a MDT that includes a psychiatrist. Patients with alcohol misuse disorders were provided with psychoeducation on the risk of postsurgical alcohol abuse and monitored for relapse following surgery [32]. Other studies in the systematic review above also addressed the monitoring of patients with PI, which is crucial for successful postoperative discharge.

Another systematic review from 2022 by Alyahya et al. examined depression after BMS and found that 27 articles including 98,757 patients reported the prevalence of post-bariatric depression. Pooling of the data revealed a prevalence of 15.3%. Severe depression was at 1.9%, moderate depression was 5.1%, and mild and minimal depression was at 12.7%. There was also a negative association between postoperative depression and weight loss (correlation -0.135), but no statistically significant association was found between post-BMS, depression, and BMI [33].

Furthermore, Morgan et al. tested the incidence and determinants of mental health service use after BMS in 24,766 patients. They found that at least one mental health service was used by 3976 patients (16.1%), with 1401 patients (35.2%) presenting only before surgery, 1025 (25.8%) presenting before and after surgery, and 1550 patients (39.0%) presenting only after surgery. An increase was seen in PIs with an incidence rate ratio (IRR) of 2.3 and emergency department attendance with an IRR of 3.0. An IRR of 2.8 for self-harm presentation, and 2.2 for drug and alcohol overdose. Furthermore, psychiatric hospitalizations had an IRR of 3.0. A total of 25 of 261 postoperative deaths (9.6%) occurred due to suicide [34]. In our study, we observed admission to a psychiatric hospital 5.1% of the time .

This shows that good pre- and postoperative assessments are necessary. Follow-up is necessary to guide and protect patients with PI in possible relapse, unstable situations, or fatal outcomes.

### Weight loss

We also tested weight loss as a secondary outcome by comparing patients with PI to a control group without PI. Postoperative %TWL was equal in both groups after six months (24.6% ± 8.9 and 24.0% ± 8.4). Steinmann et al. tested BMS in patients with bipolar and other psychiatric disorders and found similar results as in the control group after one year [12]. Another study found that BMS was a possible treatment option for stable patients with schizophrenia, with good WL results postoperative [35].

### Limitations

This retrospective study had several limitations. No validated questionnaires were used to test fluctuations in mental health, such as the Brief Symptom Inventory or disease-specific scales, such as the Beck Depression Inventory and Anxiety Symptoms Questionnaire. The Rosenberg Self-Esteem Scale was used to measure QoL. Future studies should be designed using validated pre- and postoperative measurement methods. For example, Riegel et al. deemed the dimensional DSM-5 and ICD-11 trait models suitable for defining specific “characteristics” of patients in a pre-BMS setting [36]. In addition, the patient population in this study was very heterogeneous, which provides a good representation of all psychiatric disorders and has good external validity. Nevertheless, it would be beneficial to look at a more homogeneous group to investigate the behaviors associated with individual disorders in future studies.

## Conclusion

BMS can be an effective weight loss treatment and a safe procedure for patients with psychiatric disorders. This study found no change in the psychiatric status of the patients outside their ordinary course of illness. De novo PI was rare in this study. Furthermore, patients with severe PIs were excluded from undergoing surgery and from the study. Attention to follow-up is necessary to guide and protect patients with PI.
